# The Foreign Journals: Medical Statistics

**Published:** 1839-01

**Authors:** 


### MEDICAL STATISTICS.

*View of the Operations for Lithotomy, and their Results, in the Hospital of Santa Maria di Loreto in Naples, during a period of sixteen years.* By Salvatore de Renzi.

| | Sex. | | Age. | | | Event. | |
|---|---|---|---|---|---|---|---|
| | Male. | Female. | Childr. | Adults. | Aged. | Cured. | Dead. |
| 1821 to 1836 ....... | 508 | 15 | 263 | 204 | 56 | 446 | 77 |
| Spring of 1837...... | 15 | ... | 10 | 5 | ... | 14 | 1 |
| Autumn of 1836 ... | 15 | ... | 9 | 6 | ... | 11 | 4 |
| Total......... | 538 | 15 | 282 | 215 | 56 | 471 | 82 |

This makes the proportion of deaths 1 in 6·7.

*Il Filiatre Sebezio. Dec.* 1837.

